# Impact of COVID-19 Pandemic on Stress and Burnout Levels amongst Emergency Medical Technicians: A Cross-Sectional Study in Spain

**DOI:** 10.1080/07853890.2022.2137735

**Published:** 2022-10-31

**Authors:** Tahreer Mahmoud Amro, Pedro Arcos González, Eduardo Montero Viñuales, Rafael Castro Delgado

**Affiliations:** Unit for Research in Emergency and Disaster, Faculty of Medicine and Health Sciences, University of Oviedo, Oviedo, Spain

**Keywords:** COVID-19, EMT, mental health, stress, burnout, Spain

## Abstract

**Background:**

Emergency medical technicians (EMTs) are essential health care workers (HCWs). Although they play an extraordinary role during the COVID-19 pandemic, they are mostly exposed to various occupational health and safety risks that have significantly impacted their mental health, giving rise to symptoms, such as stress and burnout.

**Aim:**

This study aimed to assess the perceived levels of stress and burnout amongst EMTs in relation to their socio-demographic characteristics and to explore the associations between their stress and burnout levels during the COVID-19 pandemic.

**Methods:**

This work is an observational cross-sectional design study conducted between 29 March and 30 April 2021, with a convenience sample of 280 Spanish EMTs yielding a response rate of 28%. The online survey had 42 items that aimed to determine participants’ socio-demographic characteristics, the Perceived Stress Scale (PSS) and the Maslach Burnout Inventory (MBI).

**Results:**

The results showed that more than half of the EMTs (53%) perceived a moderate stress level, 37% perceived moderate levels of emotional exhaustion (EE) and 40% had moderate levels of depersonalization (DP). Furthermore, 48% had low levels of personal accomplishment (PA). Gender, age, having personal protective equipment (PPE) and experiencing fear of infection were statistically significant areas where participants experienced greater stress (*p* < 0.05). A positive correlation between stress, EE and DP and a negative correlation between stress and the PA subdimension of burnout were found.

**Conclusions:**

The COVID-19 pandemic had a tremendous impact on the mental health of ambulance EMTs. Further studies building on this study and others on the psychological status of EMTs before the pandemic and follow-up during the pandemic, as well as deeper investigations on their work conditions, are needed to facilitate the implementation of various interventions. Such efforts can mitigate the negative impacts of the pandemic on their mental health, and prepare them for future disasters.

**KEY MESSAGE**
- The COVID-19 pandemic has affected the lives of the majority of the world’s population. In particular, it has impacted the mental health of various communities, including HCWs. Highly stressful and insecure work conditions have placed frontline HCWs at a high risk of psychological distress, making them victims and service providers simultaneously.

## Introduction

Amongst healthcare professionals, emergency medical technicians (EMTs) at ambulances have played an important role during the 2019 COVID-19 pandemic, working as first responders in the frontline. However, their direct contact with patients [1] exposed them to occupational health and safety risks without protection against a wide range of psychologically related consequences [2], thus leading them to experience higher severity of mental health symptoms [3,4]. Furthermore, they already face many challenges, including work overload, night shifts, irregular working hours and an extremely cautious environment that requires rapid and precise responses, all of which lead them to experience constant pressure for long periods [5]. According to a recent study, paramedics sought mental health support at almost twice the previous year’s rate before COVID-19 [6]. Moreover, the combination of persistent stress, intense emotional and work demands, insufficient ability to manage the situation, with a lack of needed resources and rewards, will create burnout as an outcome [7–9]. As a syndrome, burnout refers only to work issues and is characterized by three aspects: high emotional exhaustion (EE), high levels of depersonalization (DP) and low personal accomplishment (PA) [10]. The World Health Organization (WHO) defines ‘burnout’ as being caused by chronic workplace stress that is not managed successfully; it is listed as a disease on the International Classification of Disease (ICD-11) [11]. Past studies have found a link between this disease and the situation of ‘compassion fatigue’ amongst the frontline health care workers (HCWs) working at ambulance services, wherein they lose their ability to sympathize or engage with suffering patients [12,13]. Due to the aforementioned reasons, work-related stress amongst HCWs is considered a significant public health issue globally [14]. Previous studies have attempted to reveal the perceived levels of stress in these professionals in general [15], but no study has yet to focus on EMTs at ambulances in particular. Furthermore, although stress, depression and anxiety have been investigated, there is still a lack of data regarding burnout amongst EMTs [16]. Thus far, only a few studies have differentiated the types of burnout in the healthcare settings [17]. Moreover, most of these studies have been conducted in Asia; thus, their findings cannot be generalized due to the differences in healthcare systems and HCWs’ reactions in times of crises [18].

Hence, this study aimed to identify the correlations between EMTs’ socio-demographic characteristics and their perceived levels of stress and burnout. The study also aimed to investigate the associations between their stress and burnout levels during the COVID-19 pandemic. This work aims to provide meaningful knowledge contribution in strengthening EMTs’ psychological preparedness and improving the quality of preventive measures currently in place. It also aims to make recommendations for the provision of mental health services for EMTs, particularly during the COVID-19 pandemic and in future pandemics.

## Methodology

### Study design and setting

This is an observational cross-sectional study with a descriptive design. A convenience sample was used involving EMTs working as ambulance staff personnel (emergency and non-emergency) for the Falck ambulance company based in Barcelona, Spain. An online survey comprising 44 items was administered between 29 March and 30 April 2021. Each EMT took 3–5 min to complete the survey.

### Ethical consideration

This study was reviewed and approved by the Ethical Committee of Regional Clinical Research of the Principality of Asturias in Spain (Code No. 2021.315). Before data collection, an online informed consent form was created, which stated the purpose of this study and explained the use and protection of the participant’s personal data. The form also informed the respondents that the collection process would be performed anonymously.

### Participants

Although a total population of 1000 was targeted, only 307 managed to complete the survey in its entirety. The inclusion criteria were as follows: 1) must be an EMT; 2) must work for the Falck ambulance company in Barcelona, Spain; and 3) must be at least 18 years of age. A total of 27 respondents were excluded as they were non-EMTs. The final sample comprised 280 respondents, thus yielding a response rate of 28%). The response rate was calculated using the online Raosoft sample size method [19].

### Measurement variables and instruments

1. Socio-demographic variables: The survey asked the respondents about their socio-demographic characteristics (e.g. age, sex and education), as well as some questions regarding workload and working conditions. Additionally, they were asked about variables specific to COVID-19 (direct contact with COVID-19 patients, personal protective equipment (PPE), concerns about being infected, concerns about infecting family members, etc.).
2. Perceived stress related to COVID-19. The Perceived Stress Scale (PSS-10) was used to measure the level of stress amongst employees. This 10-item scale is widely used to measure individuals’ assessments of their stress levels [20]. In this study, we used the Spanish adaptation of the PSS-10 scale [21], which included items, such as ‘In the last month, how often have you felt nervous and stressed’? and ‘In the last month, how often have you found that you could not cope with all the things that you had to do’? These can be answered using a Likert scale ranging from 0 = ‘never’ to 4 = ‘very often’.
3. Burnout: The most widely used measurement for assessing burnout syndrome is the Maslach Burnout Inventory (MBI), which is considered the ‘gold standard’ [22,23]. The Spanish adaptation of the MBI instrument was applied in this study [24]. It has 22-items, each with seven response options on a Likert scale ranging from 0 = ‘never’ to 6 = ‘everyday’. The MBI scale also has three different components of burnout: EE: (low = <17, moderate = 18–29, high = >30); DP: (low = <5, moderate = 6–11, high = >12) and PA: (low = <33, moderate = 34–39, high = >40), where the last component is inverse, that is a high PA level means a low level of burnout, and a low PA level means a high level of burnout [22]. Sample items include the following: EE scale: ‘I feel burned out because of my work’, DP scale: ‘I am afraid that my work makes me emotionally harder’ and PA scale: ‘I deal with other people’s problems successfully’.

### Data analysis

Data were collected using Google Forms and then analysed using SPSS version 26 software (IBM SPSS; IBM Corp., Armonk, NY). Initially, the collected data were checked for missing data and outliers. Completeness was 100% amongst all included surveys, as the participants could not move on to the next question without answering the previous one. Homogeneity was checked using Levene’s test. Descriptive statistics were used, including frequencies (*n*), percentages (%), means (M), standard deviations (SDs) and minimum–maximum values. Variations between sub-categories of demographic variables were also checked. Inferential statistics approaches were used to identify differences in demographic variables: for normally distributed data, an independent sample t-test was used for comparisons of independent two groups, whilst one-way analysis of variance (ANOVA) was used for comparisons of more than two independent groups. Further, Pearson’s correlation coefficient was used to determine the relationships between variables and to establish the inter-correlation matrix [25]. The statistical significance level was set at *p* < 0.05.

## Results

Out of a total population of 1000 that was targeted, 307 completed the survey in its entirety. A total of 27 respondents were excluded as they were non-EMTs. The final sample comprised 280 EMTs, yielding a response rate of 28%. Table 1 presents the participants’ socio-demographic/descriptive characteristics. As can be seen, most of the participants were male (77% males and 23% females), the average age was 41 years old, and the highest percentage of participants’ age was for the 41–50 age group (46%). In terms of working years in Falck, we found that participants who had fewer numbers of working years (<10 years) comprised the highest percentage of the sample at 66%. Regarding weekly working hours, the results showed that more than half of the sample (54%) worked over 45 h per week during the pandemic. In terms of educational status, 212 participants (76%) had an intermediate diploma as EMTs. The questions regarding COVID-19 indicated that the vast majority of the participants (99%) have had direct contact with COVID-19 patients, and 54% stated that they did not have adequate protection measure (e.g. use of PPEs). Hence, about 45% of the participants or their family members were diagnosed with COVID-19, and 63% were afraid of getting infected/re-infected with the virus.

**Table 1.** The participants’ sociodemographic/descriptive characteristics (*n* = 280).

		Count	%
Gender	Male	216	77
	Female	64	23
Age	21–30	40	14
	31–40	73	26
	41–50	129	46
	51– 60	38	14
Years of working in Falck	≤10	186	66
	>10	94	34
Working hours per week	≤45	129	46
	>45	151	54
Highest level of education achieved	Intermediate college	212	76
	Other	68	24
Have you been in direct contact with the COVID-19 patient?	Yes	278	99
	No	2	1
During your work, do you have adequate protection measure (PPE)?	Yes	124	44
	No	156	56
Have you or any of your family member been diagnosed with the COVID-19?	Yes	128	46
	No	152	54
Do you have a fear of getting infected with the COVID-19?	Yes	176	63
	No	104	37
Occupational background	Emergency medical technician	280	100.00

Table 2 presents the comparison of the mean scale scores with the participants’ socio-demographic characteristics. The participants’ PSS scores showed that female EMTs, those from the 21–30 age group, who did not have adequate protection measure and those who had fears of getting infected with COVID-19 experienced significantly more stress than male EMTs, EMTs above 30 years old, those who had PPEs and those who did not have fears of getting infected with COVID-19, respectively (*p* < 0.05). The variables regarding number of years working in the company, weekly working hours, educational level, working in direct contact with COVID-19 patients and were diagnosed with COVID-19 (or their family members), none of them had a statistically significant difference (*p* > 0.05). Meanwhile, after comparing the participants’ mean scores from the MBI EE subdimension, significant differences were found in terms of gender, age, having PPEs and fear of getting infected with COVID-19 (*p* < 0.05). In particular, females experienced higher EE than males, and those in the 31–40 age group had the highest EE. Furthermore, the participants who did not have PPEs and had fears of being infected with COVID-19 had higher mean scores in EE than those who had adequate protection measures and those who were not afraid of infection.

**Table 2.** Comparison of the mean scale scores with the participants’ characteristics (*n* = 280).

		Perceived Stress Scale (PSS)			Maslach Burnout Inventory (MBI)								
					EE			DP			PA		
Variables	*N*	Mean	SD	*p*	Mean	SD	*p*	Mean	SD	*p*	Mean	SD	*p*
Gender													
Male	216	16.52	8.04	**0.011**	24.23	10.81	**0.039**	8.97	5.40	0.767	33.35	7.23	0.709
Female	64	19.42	7.72		27.48	11.81		9.20	5.72		32.97	7.10	
Age													
21–30	40	20.25	6.08	**0.002**	25.50	9.98	**0.021**	9.95	4.55	0.597	32.75	5.14	0.172
31–40	73	18.07	8.53		27.04	11.45		9.26	5.72		31.82	8.35	
41–50	129	16.82	8.31		25.05	11.30		8.74	5.78		33.98	6.65	
51–60	38	13.47	6.59		20.18	9.80		8.58	4.76		34.16	8.14	
Number of years working in Falck													
≤10	186	17.81	7.780	0.066	25.19	11.054	0.644	9.20	5.711	0.455	33.44	6.941	0.564
>10	94	15.94	8.466		24.54	11.257		8.68	4.952		32.91	7.686	
Working hours per week													
≤45	129	17.38	7.22	0.701	24.57	9.95	0.564	8.57	5.18	0.202	33.14	6.79	0.789
>45	151	17.01	8.72		25.32	12.03		9.41	5.68		33.37	7.54	
Highest level of education achieved													
Intermediate college	212	17.11	7.82	0.801	24.90	10.84	0.844	9.21	5.57	0.325	33.38	6.89	0.657
Other	68	17.40	8.79		25.21	11.99		8.46	5.12		32.90	8.09	
Have you been in direct contact with a COVID-19 patient?													
Yes	278	17.14	8.06	0.349	24.94	11.11	0.564	9.05	5.46	0.361	33.28	7.21	0.728
No	2	22.50	6.36		29.50	13.44		5.50	7.78		31.50	3.54	
During your work, do you have adequate protection measure (PPE)?													
Yes	124	15.55	7.14	**0.002**	21.88	10.60	**0.000**	8.91	5.54	0.757	33.73	7.26	0.339
No	156	18.48	8.51		27.44	10.91		9.12	5.42		32.90	7.14	
Have you or any of your family member been diagnosed with COVID-19?													
Yes	128	17.21	8.52	0.956	23.99	11.34	0.175	8.80	5.11	0.537	33.50	7.75	0.620
No	152	17.16	7.66		25.80	10.88		9.21	5.76		33.07	6.71	
Do you have a fear of getting infected with COVID-19?													
Yes	176	18.64	7.83	**0.000**	26.85	10.59	**0.000**	9.14	5.23	0.658	32.77	7.08	0.132
No	104	14.72	7.86		21.81	11.29		8.84	5.86		34.11	7.33	
Occupational background													
Emergency medical technician	280	17.18	8.05	**0.000**	24.98	11.11	**0.000**	9.03	5.46	**0.000**	33.26	7.19	**0.000**

Data were statistically significant (*p* < 0.05) are bold.

T-test, one-way ANOVA, Tukey post-hoc test and Scheffe test.

Meanwhile, the mean scores of the remaining variables: number of working years at the company, working hours per week, educational level, direct contact with COVID-19 patients, and diagnosed with COVID- 19 were also compared, with the results indicating no significant differences regarding EE as an element of burnout scale (*p* > 0.05). Moreover, the participants’ mean scores in the DP and PA subdimensions were compared, and the results showed that none of the socio-demographic variables were significantly associated with these two subdimensions (*p* > 0.05).

Table 3 reveals the correlation between stress and burnout amongst all the participants (*n* = 280). The results showed a moderate positive correlation between the perceived level of stress and burnout amongst EMTs (0.49), wherein the higher the stress, the higher the burnout. Specifically, the findings showed a strong positive correlation between stress and EE as well as a moderate positive correlation with DP (0.62) and (0.48). However, there was a moderate negative correlation between the level of stress and the third subdimension of burnout (PA: 0.45), where the increase in the stress level correlated with a decrease in the PA amongst EMTs.

**Table 3.** Correlations between stress and burnout amongst EMTs (*n* = 280) (Pearson correlation).

		EE	DP	PA	Burnout
Stress	Pearson correlation	0.62**	0.48**	–0.45**	0.49**
	Sig. (2-tailed)	0.000	0.000	0.000	0.000
	*N*	280	280	280	280
Correlation interpretation		Strong positive	Moderate positive	Moderate negative	Moderate positive

The ** according to the Pearson's correlation interpretation: Strong Positive Correlation= 0.5–0.7; Moderate Positive Correlation= 0.3–0.5; Weak Positive Correlation= > 0–0.3; Weak Negative Correlation= < 0–-0.3; Moderate Negative Correlation= -0.3– -0.5; Strong Negative Correlation= -0.5– -0.7. https://www.slideshare.net/phannithrupp/guideline-for-interpreting-correlation-coefficient

**Figure 1.**
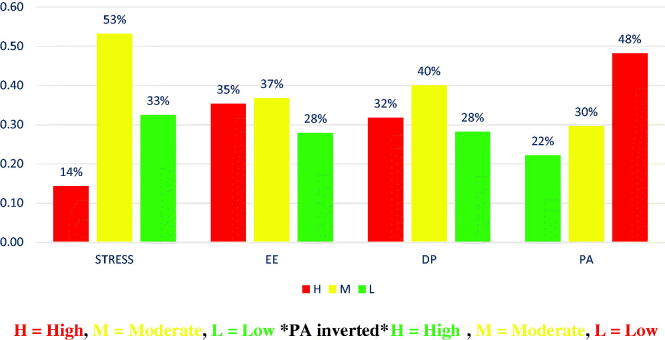
PSS and MBI Scale scores amongst EMTs (*n* = 280) (2021).

Figure 1 demonstrates that over half of the participants (53%) perceived moderate stress levels, whilst 14 and 33% perceived high and low levels of stress, respectively. For the burnout scale, the highest percentage of participants (37%) perceived moderate levels of EE, followed by 35% and 28% who had high and low levels of burnout, respectively. Meanwhile, moderate levels of DP were perceived by the highest percentage of participants 40%, whilst high and low levels of DP were experienced by 32 and 28% of participants, respectively. However, regarding the PA subdimension of burnout, only 22% of all participants had high PA levels, whilst 30 and 48% had moderate and low PA levels, respectively, where the scale of this subdimension was inverted: a high level of PA indicated low level of burnout and a low level of PA indicated a high level of burnout). Therefore, in this study, the highest percentage of participants who had low levels of PA also had high levels of burnout according to the MBI Scale [22].

## Discussion

According to the study, being an EMT in general, working during a serious pandemic (e.g. COVID-19), the lack of PPEs, having fears of getting infected with the virus, being female and belonging to the young to middle age group, could be risk factors in developing stress and burnout. Multiple studies have shown that these same factors were the main causes of developing psychological symptoms amongst frontline health workers [17,26]. Gender and age showed statistically significant differences in this study in terms of stress. Female EMTs and those in the 21–30 age group had higher stress levels than males and older EMTs. These findings are supported by the literature, which found high-stress levels amongst females and young HCWs [27]. According to another study, young healthcare professionals and those who had just started their careers had higher stress levels [28]. The significant difference in the stress levels of young and female workers is expected. It is a well-known medical fact that the hormone system in women makes them more vulnerable to psychological distress [29]. Furthermore, young age is usually characterized by less experience of dealing with obstacles and mental pressure in various fields [30].

In addition, having adequate PPEs had a statistically significant impact in terms of differences in stress levels. In this study, the stress levels of EMTs who did not have access to proper PPEs during work were higher than those who used PPEs, in accordance with the literature. The government of Spain reported that, by the end of March 2020, around 9444 HCWs were affected by the shortage of PPE [31]. The availability of PPEs provides frontline HCWs with a safer feeling against infection, which in turn, relieves their stress [32]. Thus, a shortage in supplies can exacerbate stress levels among HCWs, as the lack of PPE, playing an essential role in contracting and transmitting the disease during epidemics [33].

Moreover, having a fear of COVID-19 was found to be significantly associated with a higher level of stress. In particular, the *p* value was 00.00, which indicated a genuine difference, in line with previous studies. According to a previous study, around 1153 Italian frontline HCWs had higher levels of stress due to the heightened risk of getting infected whilst working exclusively at COVID-19 wards [34]. In this study, EMTs faced an unusual, dangerous disease that has afflicted the whole world for the first time with insufficient PPEs. This is a major reason why they are experiencing stress.

The variables female gender and age group (31–40 years) had significant correlations with EE levels. This is in accordance with a previous study, which found that women had higher levels of EE than men [35]. Indeed, EMTs from the 31–40 age group most significantly suffered from a tiring, stressful and difficult relationship with their work, which were symptoms of EE, in line with the definition of ‘work-related’ burnout provided by the WHO: a phenomenon caused by chronic stress in the workplace not managed successfully [11]. Given that EMTs in the younger age group might experience stress but for a relatively shorter period of time, they may not have yet developed a high level of burnout.

As for not having PPE and having fears of infected, both results were significant and similar to the previous studies. In the context of a pandemic, the insufficiency of PPEs is considered a major stressor that increases burnout amongst HCWs [36]. A study reported that HCWs who worked with COVID-19 patients had high levels of fear and poor well-being [37]. Thus, it makes sense that working as a frontline HCW during a pandemic whilst dealing with suspicious and confirmed cases of a highly contagious disease without a PPE would make any individual afraid of getting infected. In turn, due to the state of being in fear for a prolonged period of time, HCWs are likely to develop exhaustion symptoms whilst performing their jobs, thereby leading to occupational burnout. The remaining socio-demographic variables were compared in terms of perceived stress and EE, but the results did not show any statistically significant difference. Furthermore, none of them had a statistically significant correlation with the DP and PA subdimensions of the MBI Scale.

Conducting Pearson correlation test between perceived stress and burnout in general and between stress level and the three elements of burnout revealed significant correlations in the expected directions, wherein a positive moderate correlation between the perceived level of stress and burnout amongst EMTs was found. In previous studies on other pandemics, authors found that burnout was associated with stress amongst frontline health workers [38]. Furthermore, perceived stress had a positively moderate correlation with DP and a negatively moderate correlation with PA. On the other hand, a strong positive correlation between stress and EE was found. This is a highly expected outcome, as it followed the medical interpretation stating that stress leads to burnout especially in working conditions where the variables compared in terms of stress showing statistically significant differences were the same variables having statistically significant differences in terms of EE. The negative correlation between perceived stress and the third subdimension of burnout can be attributed to the fact that PA has an inverted scale, wherein a high score means an individual’s positive view of his/her PA at the workplace and a low score indicates poor well-being in the same situation. Here, stress is associated with EMTs having moderately low PA during the COVID-19 pandemic, which echoes the finding of another study conducted in Spain, which found that stress is negatively associated with PA amongst Spanish health personnel during COVID-19 outbreak [10].

As an overall finding of this study, more than half of the EMTs (53%) perceived having moderate stress levels, 37% of them perceived having moderate levels of EE, 40% had moderate levels of DP and 48% had low levels of PA during the COVID-19 pandemic. The moderate levels of stress and the first two subdimensions of burnout are not on the same line with literature, which reported significantly high levels of stress and burnout amongst frontline HCWs in Italy whilst working with COVID-19 patients [38] and high levels of stress and burnout amongst frontline HCWs in Turkey [39]. These findings could be due to the fact that the majority (77%) of the participants were males. In addition, this could be due to the educational level attained by the EMTs, where 212 out of 280 graduated with an intermediate diploma. Since 2007, a two-year unified intermediate occupational diploma has been adopted throughout Spain under the ‘Emergency Medical Technician’ programme at vocational-community colleges, including specialized training that leads to highly qualified EMTs [40]. This has been revealed by a study, which explained that the main factor for burnout is the gap between the level of existing skills and the level of skills needed in the workplace typically characterized by high levels of pressure and chronic stress; thus, highly qualified workers have greater control and less burnout [41].

At the same time, the findings of the overall sample showed that a high percentage of EMTs in the study (almost half of the sample) suffered from low levels of PA, in accordance with the literature. A study conducted in Belgium revealed the prevalence of low PA amongst frontline HCWs during the COVID-19 pandemic [18]. However, another study revealed that emergency staff working at COVID-19 wards had low levels of burnout based on their high PA levels, which can be attributed to their feeling of being given more value whilst working in such a risky ward [41]. PA, which is defined by Maslach as the ‘safety valve’ that balances the situation when EE and DP occur, refers to the high self-efficacy and feeling of achievement, which when reduced, usually indicates burnout [22]. In this study, which revealed the moderate levels of EE and DP amongst EMTs, we can conclude that the EMTs were able to complete their work and maintain their professional obligations; however, such obligations may be a stressor to the EMTs, which may affect them personally and be detrimental to their mental health [42]. According to Maslach, the low PA level among many EMTs (regardless of the EE and DP levels), expresses the degree of burnout. This is a problem requiring urgent attention, as it indicates a negative psychological impact on EMTs and prevalent negative views of their PA in the workplace. Such a negative psychological status is a cornerstone to realizing how burnout affects their mental status and its connection to work under such high-pressure conditions. In fact, multiple studies have recently reported that the numerous cases of suicide amongst frontline HCWs are related to work conditions during the COVID-19 pandemic [43,44].

Mental health support during and even outside work is strongly advised, starting from the presence of psychological counselling at the workplace, with the possibility of communication outside of working hours and workplaces. Furthermore, there should be efforts to hold periodical mental health meetings, workshops and training on psychological self-care. Such interventions can be conducted by the occupational health services departments, which can carry out regular monitoring programs, in addition to stress risk assessment, risk management and workplace mental health promotion programs for frontline HCWs [45]. It would also be beneficial to adjust working hours and weekly workloads, improve the work environments and make them healthier by providing EMTs more time to rest, exercise and even shower during working hours [46]. They can also be given opportunities to strengthen their spiritual health at work, because reviving one’s spiritual side has been shown to generate mental health benefits [47]. Moreover, one of the fundamental preventive interventions that can be a major factor affecting one’s sense of assurance and consequently improve psychological symptoms is the availability of PPE and prioritization of frontline EMTs in the vaccination process to the maximum extent possible [48]. Such efforts can contribute to building the psychological resilience of EMTs, which in turn, will mitigate the impacts of the COVID-19 pandemic and make them more prepared for future disasters.

## Limitations

First, with a cross-sectional study design, it was not possible to determine the causality between exposure and outcome as they were assessed at the same time; thus, it cannot provide information on the development of the outcomes over time. Moreover, the stress and burnout of participants before the study period were not recorded. Second, due to the sampling method, the sample included only participants from one organization (Flack’s Barcelona branch). Thus, the EMTs presented their own ways of expressing their feelings of stress and burnout; thus, the findings cannot be generalized to other contexts. Finally, a recall bias may be present as the participants were asked to answer questions about their situations from the previous month. The questionnaire was a self-report evaluation questionnaire, which did not consider a proper diagnostic method.

## Conclusions

The perceived moderate levels of stress and burnout might increase in the near future if not addressed properly. The mental health status of frontline HCWs is very crucial and has an influence on their personal achievements, the quality of the service that they provide and their overall work satisfaction. All of these can lead to a high number of resignations amongst EMTs. However, the harm may continue, as staying at work affects them, whilst quitting will affect their colleagues due to the potentially increased workload and high demand due to the pandemic. Therefore, policymakers and health administrators should be aware of the health workers’ mental health and how work-related conditions can influence this. The authorities must also recognize and address the factors associated with stress and burnout symptoms in EMTs as well as implement preventive organizational strategies appropriately. Therefore, it is very important to work on further research building on the findings of this study and others on the psychological status of EMTs before the pandemic, conduct follow-up during the pandemic, and carry out in-depth investigations on the work conditions of EMTs. In this way, we can gain a comprehensive view that facilitates the urgent implementation of interventions that can mitigate their stress and burnout levels and support their overall mental health.

## Data Availability

All relevant data are included in the article. Additional supporting data are available from the corresponding authors upon request. All requests for raw and analysed data and materials will be reviewed by the corresponding authors to verify whether the request is subject to any intellectual property or confidentiality obligations.
